# Deep-learning-based polarization-dependent switching metasurface in dual-band for optical communication

**DOI:** 10.1515/nanoph-2025-0370

**Published:** 2025-11-18

**Authors:** Yihan Yan, Yunkai Wu, Yangwen Wang, Jiahao Li, Jingtian Hu, Xu Wang

**Affiliations:** College of Big Data and Information Engineering, 71206 Guizhou University, Guiyang, 550025, China; Guangdong Provincial Key Laboratory of Semiconductor Optoelectronic Materials and Intelligent Photonic Systems, Harbin Institute of Technology, Shenzhen, 518055, China; Key Laboratory of Advanced Manufacturing Technology, Ministry of Education, Guiyang, 550025, China

**Keywords:** deep learning, metasurface, polarization-dependent switching, dual channel, optical communication

## Abstract

To address the critical limitations of conventional band-switching technologies – such as their slow speed, high energy consumption, and mechanical instability – this research introduces a novel deep-learning-driven framework for the intelligent inverse design of polarization-multiplexed metasurfaces. This approach represents a paradigm shift from traditional methods by enabling single-step, computational discovery of metasurface designs that directly encode two distinct optical functions within a single flat device. At the heart of our framework is a custom-designed deep neural network that seamlessly integrates parallel convolutional layers for robust feature extraction with cascaded regression modules for high-precision prediction. This hybrid architecture allows us to engineer sub-wavelength meta-atoms to achieve desired optical responses rigorously. As a groundbreaking demonstration, we designed and optimized a metasurface that achieves dynamic band switching solely through polarization modulation: it generates a targeted transmission peak in the O-band (1,260–1,360 nm) under *y*-polarization and an independent peak in the C-band (1,530–1,565 nm) under *x*-polarization. This mechanism eliminates the need for moving parts. The resulting device exhibits a switching efficiency orders of magnitude greater than its mechanical counterparts, while simultaneously offering enhanced stability, lower power consumption, and inherent adaptability for reconfigurable optical networks. Our work not only validates a specific device but also establishes a robust and generalizable design paradigm, underscoring the transformative potential of uniting deep learning with metasurfaces to achieve ultra-fast, intelligent, and efficient photonic systems for next-generation optical communications.

## 1 Introduction

With the rapid development of intelligent optical systems [1] and communication technologies [2], the ability to precisely tailor the wavelength of the optical signal is becoming increasingly crucial for applications ranging from wavelength-multiplexing in 5G to hyperspectral imaging. Traditional wavelength switching methods usually depend on physical devices, such as mechanical optical switches [3], fiber optic splitters [4], [5], and thin film interference filters [6], [7], [8] which suffer from low processing speed, large device size, and limited robustness, prohibiting their applications in next-generation technologies towards superb operation bandwidth and miniaturization [9]. Existing techniques also exhibit complex device setups that require post-fabrication tuning while lacking the versatility and tunability for high-frequency, fast band switching [10]. Therefore, improving the speed, precision, and flexibility of band switching has become a crucial task in the present field of optical communications.

With the rapid development in nanophotonics [11], metasurfaces – artificially structured electromagnetic surfaces with advantages of ultrathinness [12], compactness and the ease of on-chip integration – have enabled versatile and flexible wavefront modulation capabilities [13], [14], [15] in all degrees of freedom of light including amplitude [16], [17], [18], [19], phase [20], [21], [22], [23], [24], and polarization [25], [26], [27], [28], [29]. Recent advances in deep learning (DL) based on artificial neural networks further provide a powerful tool for universal design of metasurfaces involving simultaneous optimization of their unit geometry, materials composition, and spatial arrangements, enabling information encryption [30], [31], [32], holographic display [33], [34], [35], and optical communication [36], [37], [38]. These computational methods allow researchers to rapidly optimize metasurface designs in a data-driven manner, dramatically improving the possibility of information capacity. For example, Parka et al. [39] proposed high-capacity single-cell metasurface capable of maximizing channels by multiplexing holographic images across both spin and wavelength using a single-phase map. Further, Pierangeli et al. [40] proposed a new method for transmitting full Stokes polarization images through a scattering medium using DL, where any input polarization image can be reconstructed in a single shot using only an intensity sensor. Zhang et al. [41] proposed a DL-based data iteration strategy that enables a specially designed metasurface to explore an almost infinite chiral metasurface design space. Although these studies have made some progress in the field of optical metasurface modulation by significantly expanding the number of channels on the metasurface through a variety of methods, thus dramatically enhancing the data transmission capability of a single metasurface, they have neglected how to utilize the metasurface to achieve efficient switching between different information channels.

In recent research, band-switching metasurfaces have achieved significant progress in both microwave and terahertz bands. For instance, Sun et al. [42] and Zhang et al. [43] demonstrated functional switching between optical and microwave signals, while Wang et al. [44] employed spatial coding techniques to achieve wavefront manipulation in the microwave band. On the other hand, research attention has expanded to the frequency-domain switching of transmission peaks, as demonstrated by Pattanayak et al. [45] achieved switching of transmission peaks under orthogonal polarization in the terahertz band using asymmetric aperture arrays; Farooq et al. [46] dynamically realized switching between C-band and X-band frequency-selective surfaces via diode switches. However, the work mentioned above has concentrated on the microwave or terahertz frequency bands. In optical communication bands (such as the O-band, C-band, and L-band), achieving pure transmission peak position switching remains an under-explored technological frontier.

Here, we present a deep-learning-enabled approach to metasurface design, enabling efficient switching of communication bands. The metasurface ingeniously utilizes the polarization-dependent electromagnetic response properties: in the case of *x*-polarization, the device exhibits efficient transmission peaks in the C-band, while in *y*-polarization, significant transmission peaks are seen in the O-band. The design combines the selection of the communication band with the control of the polarization state of the light source and is able to optimize the conventional band-switching scheme in terms of integration, response speed, and control complexity. By combining DL technology with inverse design and optimizing the metasurface structure through neural network model training, the metasurface structure can satisfy the switching requirements of specific bands in the shortest time, thereby effectively improving efficiency. We also performed a comprehensive optical analysis of the designed metasurface, including electromagnetic field distribution simulations, transmission spectral line properties, and coupling mechanism studies in different polarization states. By deeply exploring its working principle, a solid theoretical foundation has been laid for the performance verification and further optimization of the metasurface. The results show that this novel metasurface is capable of accurately controlling the switching of two communication bands, which provides the necessary technical support for the fiber optic channel in modern optical communication systems. It is expected to significantly improve the system efficiency in multiwavelength multiplexing (WDM) systems to enhance their capacity and optimize spectrum utilization, providing a new perspective for the future development of optical communication technology.

## 2 Materials and methods

### 2.1 The iterative process of polarization-dependent switching metasurface

**Figure 1:**
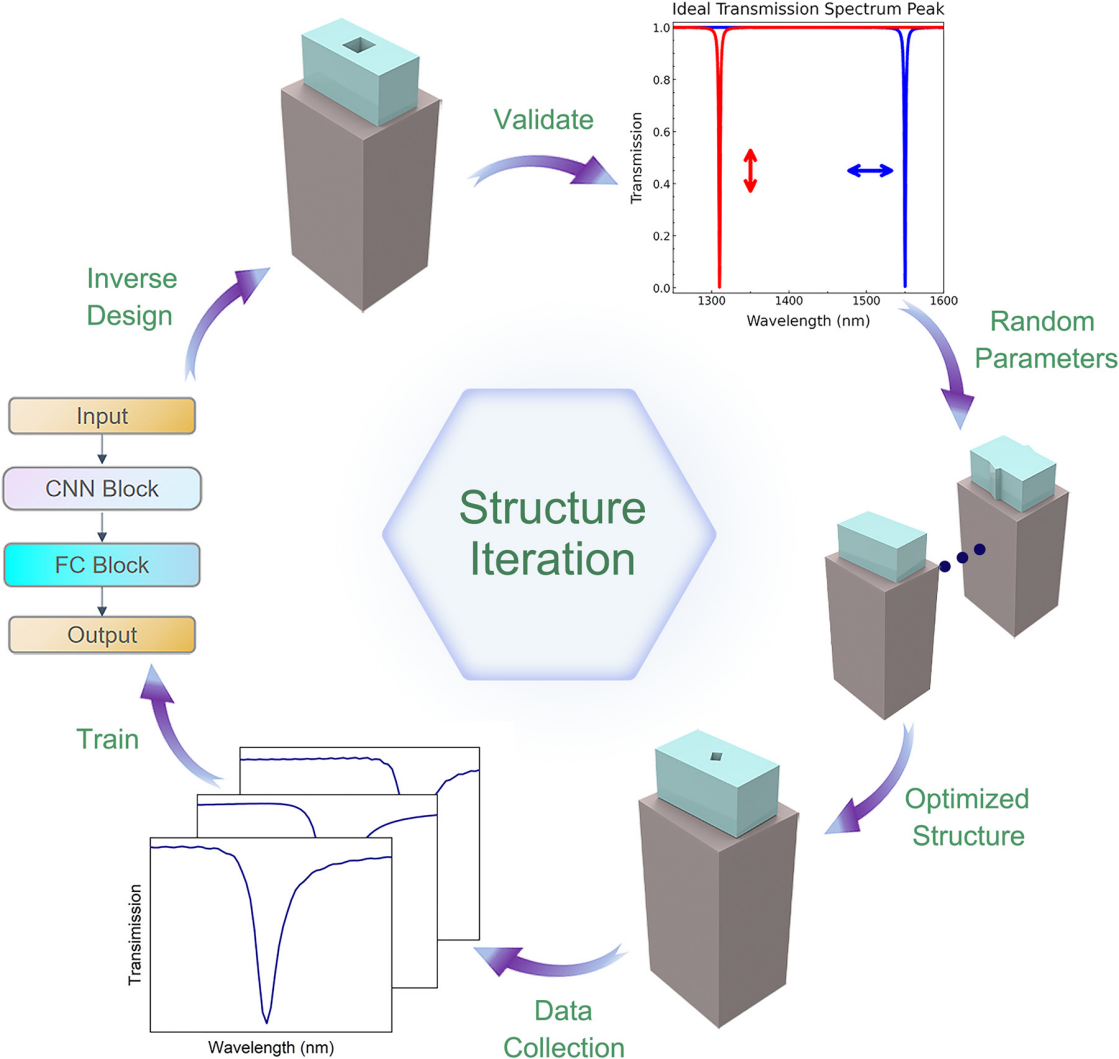
Iterative diagram of the metasurface structure. The iterative process starts with generating the initial structure by random parameter search and screening the structure to introduce holes for tuning and optimization. Subsequently, the data are collected and fed into the neural network for training, and finally, the desired structure is obtained in reverse.

The metasurface optimization employs an iterative design process (Figure 1) that combines electromagnetic simulation with data-driven deep learning techniques. This inverse design approach uses a result-oriented search to rapidly identify the optimal metasurface design that achieves our targeted spectral responses. To enable this data-driven training process, we prepared a library of 3,000 meta-unit structures with simulated spectral responses calculated by Lumerical FDTD [13] with a Python interface. These simulations were all performed under periodic boundary conditions in the *x* and *y* directions with a periodicity of 1,076 nm and 906 nm, while a perfectly matched layer (PML) boundary condition was applied in the *z-*direction.

The initial screening process for metasurface structures is significantly time-consuming, relying on manual searches and empirical judgments. Through systematic manual simulation and spectral analysis, we progressively evolved the TiO_2_ cross-shaped structure on a SiO_2_ substrate into a complete nano-block with a nano-hole. Ultimately, we identified a structure capable of achieving polarization-dependent transmission peaks in both the O-band and C-band, establishing it as the initial configuration. TiO_2_ is ideally suited as the primary material for metasurface atomically-scale structures to enhance light modulation due to its relatively high refractive index and low optical loss. The relevant material parameters are based on TiO_2_ (Titanium Dioxide) - Sarkar. To provide stable support and ensure the structure’s mechanical stability, SiO_2_ material is used as the substrate with relevant material parameters based on SiO_2_ (Glass) – Palik.

This class of nanophotonic structures was selected because of their significant polarization-dependent optical responses that provide greater control over doubly polarized light bands. In addition, the extra nano-hole in the top nanopillar units further allows the fine-tuning of wavelength- and polarization-dependent responses of these devices through optimization of the shape and size of the holes. By rationally tuning the angle and size of the holes, the transmission performance can be improved in a specific wavelength range, leading to effective localization of light by the structure and thus more efficient control of optical power and its dispersion over wavelengths. Since the preliminary design scheme was obtained by manual screening, the process shows considerable potential for improvement in terms of efficiency and accuracy, so that optimization using deep learning methods remains essential in the next step. We trained the structural parameters of the metasurface and the transmission spectral data obtained from the simulation as the input and output of the neural network at the same time, where the rapid decrease of the loss function from 1.35 towards 0.65 indicated that the neural network could capture the relationship between the structural parameters and the optical response. By learning the correspondence of a large amount of data entered, the neural network could effectively search for the optimal solution in the structural parameter space and thus accurately reverse-engineer the metasurface structure to meet the specific requirements. Compared to traditional manual tuning or numerical optimization, our approach exhibits superior efficiency and accuracy in realizing the optimal desired targeted optical responses in a complex design space.

### 2.2 Structure optimization process

#### 2.2.1 Data set acquisition

**Figure 2:**
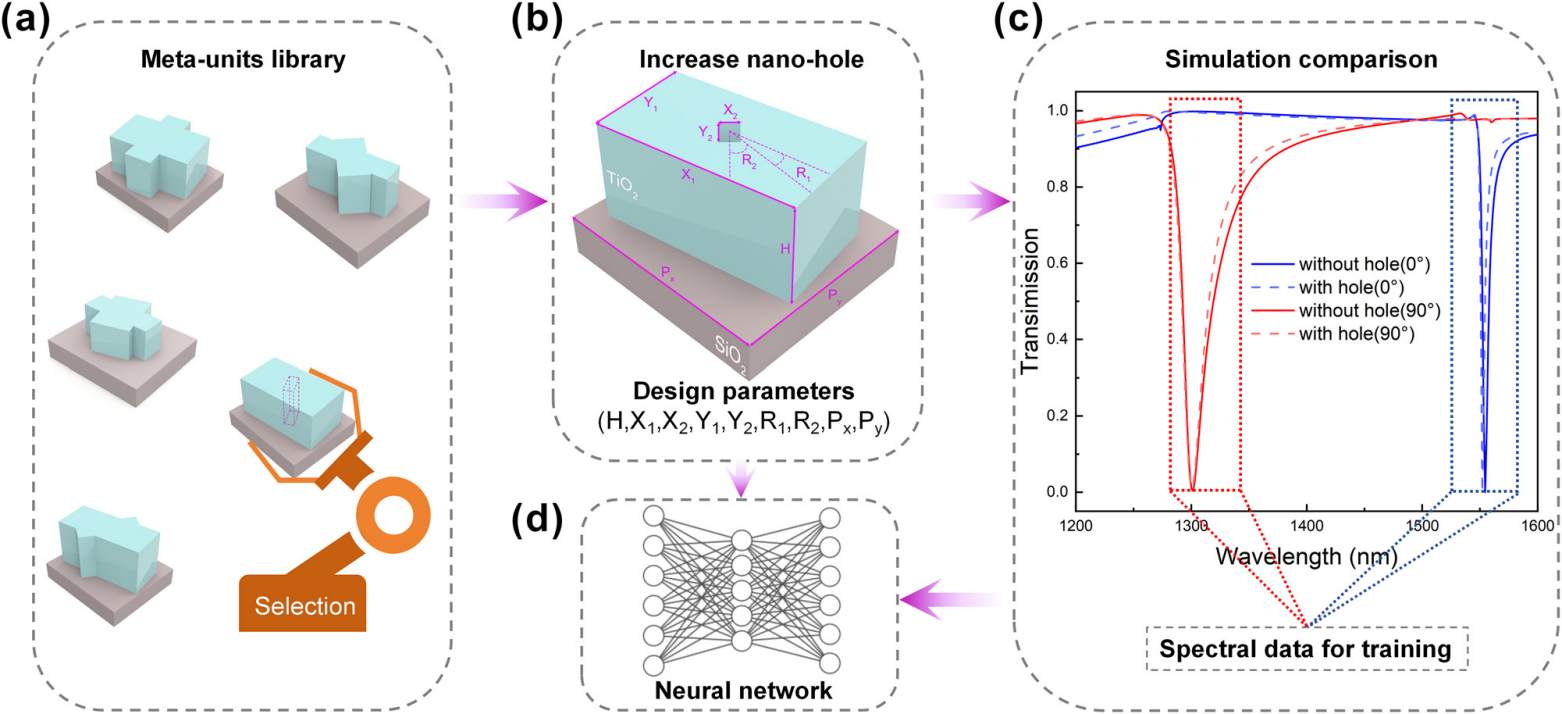
Deep learning data acquisition diagram. (a) Meta-atom library with various metasurface unit structures generated by different random parameters, and selecting one as the initial structure. (b) Nine structure parameters are used as the training dataset for increasing nano-hole structures. (c) Compare spectral curves with and without a nano-hole. Spectral data from the red dashed line (wavelength range 1,280–1,340 nm) and the blue dashed line (wavelength range 1,520–1,580 nm) were extracted as the training dataset. (d) Schematic diagram of the neural network.

Figure 2 illustrates our strategy and methodology for preparing the training dataset, enabling a data-driven neural-network training process that ultimately leads to optimized structure and data processing. In the metasurface unit structures generated by different random parameters, as shown in Figure 2(a) “Meta-units library,” most structures can be regarded from a top-down perspective as a rectangular nanoblock joined at the top and bottom with two additional triangular or two additional trapezoidal nanoblocks. Evidently, such structures are highly complex and challenging to fabricate. Notably, a simple structure was identified where smaller nanoblock particles are covered by larger ones, resulting in the appearance of a single nanoblock. The spectral response of this structure enables independent transmission peaks in the C-band and O-band under different polarizations, as shown in Figure 2(c). Because its simple structure and spectral response exhibits a dual-band transmission peak design requirement, we selected this structure as the initial structure and introduced the nano-hole into it as a further optimization step. Considering that the previous structure was obtained by rotating two nanopillars around the center, where the central hole was analogously treated as an air block (refractive index *n* = 1.0) undergoing rotation, the thought process leads to the structure shown in Figure 2(b). By tearing the in-plane orientation of this small air cube and the large rectangular nano-block, the period *P*_*x*_ in the *x*-direction of the metasurface cells, the period *P*_*y*_ in the *y*-direction, the width *X*_1_, the length *Y*_1_, the rotation angle *R*_1_ of the TiO_2_ nano-block, the width *X*_2_, the length *Y*_2_, the rotation angle *R*_2_ of the air rectangle, and the heights *H* of both, a total of nine different structural parameters were used as the training dataset.

The tuning ranges of these structural parameters, as shown in Table 1, cover sufficient design space that ensures the desirable spectral responses can be realized by our metasurface, providing diverse input data for subsequent deep-learning training. To verify that the presence of holes will have a positive effect on the optical response (i.e., higher *Q* value) of the metasurface, we simulated and compared the two structures separately, and the relevant comparison effects are shown in Figure 2(c). The performance characterized by the transmission spectrum of the structure with and without holes was compared, indicating that the meta-unit with holes exhibited a substantial reduction in the spectral linewidth, which closely matched our design objective and confirmed the rationality of our holey unit-cell designs.

**Table 1:** Range of variation of structural parameters.

Name of parameters	Scale of parameters
*H*	400 nm∼500 nm
*X*_1_	800 nm∼1,000 nm
*X*_2_	100 nm∼300 nm
*Y*_1_	400 nm∼500 nm
*Y*_2_	100 nm∼300 nm
*R*_1_	−10°∼10°
*R*_2_	−45°∼45°
*P*_*x*_	1,000 nm∼1,100 nm
*P*_*y*_	850 nm∼950 nm

To facilitate effective convergence of the neural-network model during training, we adjusted the sampling of our training data by selectively focusing on the two spectral bands that covered our design objectives so that the exploration of the design space with extremely high input-output dimensionality could be more effective. Specifically, the training data were collected with increased sampling frequency on the two wavelength ranges of interest: *λ* = 1,280–1,340 nm and 1,520–1,580 nm. This strategy reduced the spectral input per structural parameter set to a total of 120 points. Consequently, each group of 9 structural parameters in the dataset corresponds to 120 spectral points, effectively reducing the data’s dimensionality and simplifying the training process of the neural network. Eventually, all the datasets were collected and organized into 3,000 sets of data, of which 80 % was used as the training set and 20 % as the validation set.

#### 2.2.2 Forward and inverse neural networks

**Figure 3:**
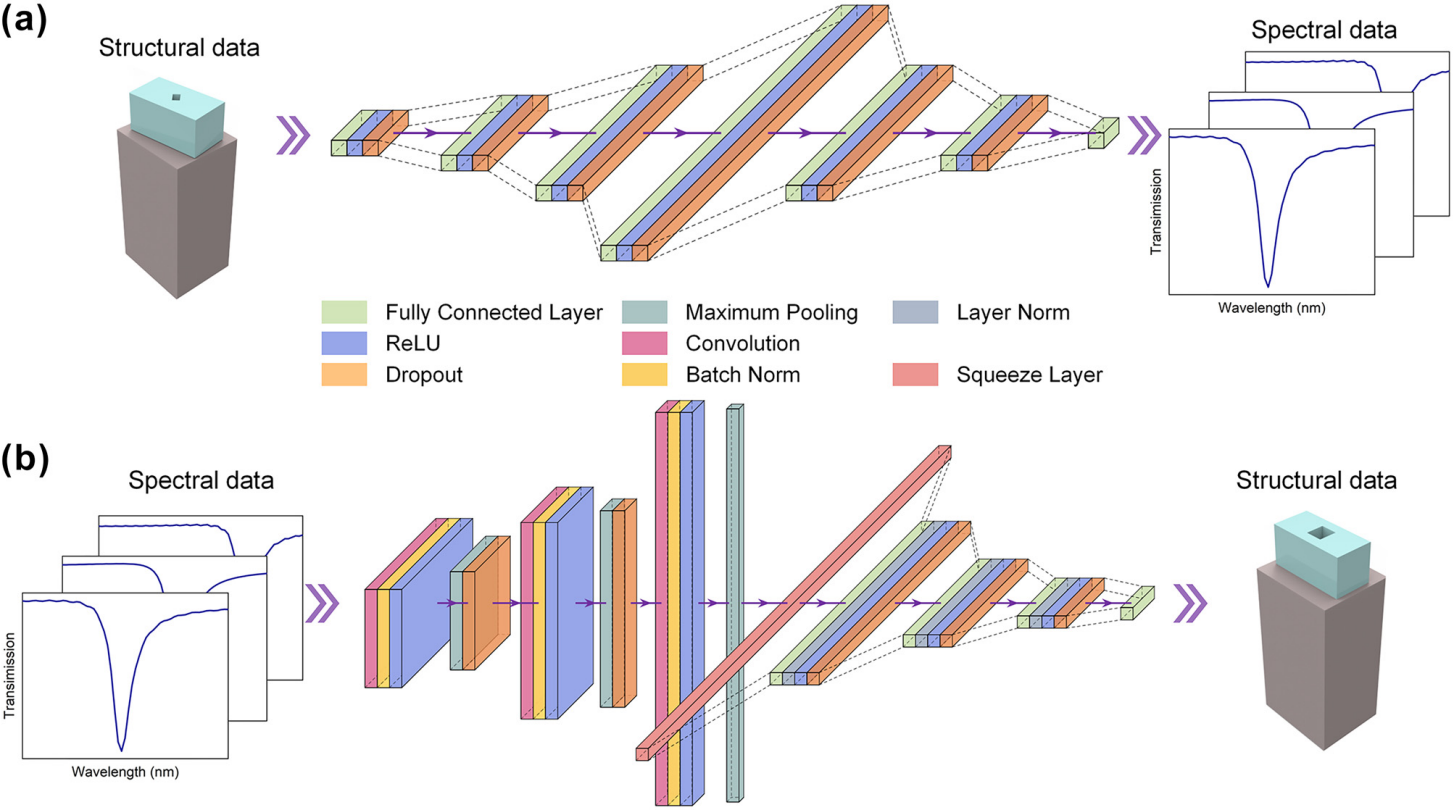
Schematic structure of forward and inverse neural networks. (a) Forward network, predicting spectral data from structural parameters. (b) Inverse network, predicting structural parameters from spectral data.

We further optimized our neural network architecture to enhance the efficiency of our structural optimization. We developed a pair of neural networks where Figure 3(a) shows the first one, called the forward neural network, can effectively predict the spectral response of any metasurface designs, and Figure 3(b) the second network, called the inverse neural network, was trained using the forward model to perform inverse design of metasurfaces based on the desired spectral responses. As shown in Figure 3, which depicts the network architectures, the longitudinal length of each rectangle is the number of neurons in that layer, the height is the number of channels, and the width is the dimension size. The forward neural network model, depicted in Figure 3(a), employs a multilayer perceptron (MLP) architecture [47]. This model is designed to predict 120 spectral data points from 9 structural parameters (*H*, *X*_1_, *X*_2_, *Y*_1_, *Y*_2_, *R*_1_, *R*_2_, *P*_*x*_, *P*_*y*_) through deep learning. The nine neurons in the network’s input layer correspond to the number of structural parameters. Then, the hidden layer of the model gradually adopts the form of an ANN network that gradually widens and then narrows [48]. The output layer consists of 120 neurons, matching the dimensionality of the spectral data. In such architectures, the symmetric expansion and reduction of the hidden layers facilitate comprehensive learning of high-dimensional input data features during the initial stages [49]. Deep within the hidden layers, a decrease in the number of neurons allowed the network to refine the features to avoid overfitting while obtaining the kernel weights and bias for optimal feature extraction. In addition, a module consisting of a fully connected layer (green rectangle in Figure 3), a ReLU activation function (blue rectangle), and a dropout (orange rectangle) provided the necessary nonlinearity to capture complex relationships in the data as well as the reduction of overfitting phenomena, ensuring that the model has a stronger generalization capability. The detailed input and output dimensions of the forward prediction model are shown in Table 2.

**Table 2:** Forward neural network dimensions input and output table.

Block	Size-in	Size-out
FC block 1	Input size	512 × 1
FC block 2	512 × 1	1,024 × 1
FC block 3	1,024 × 1	2,048 × 1
FC block 4	2,048 × 1	4,096 × 1
FC block 5	4,096 × 1	2,048 × 1
FC block 6	2,048 × 1	1,024 × 1
FC layer	1,024 × 1	Output size

We used a forward network to make an initial attempt to observe the correlation between structure and spectral data. Considering the code compatibility, we adopted the Adam optimizer, which combines momentum and adaptive learning rate mechanisms, and the CyclicLR learning rate scheduler capable of dynamically adjusting the learning rate during the training process. For the loss function design, combining gradient-sensitive weights with minimum-sensitive weights enables dynamic adjustment according to the target data’s gradient changes. This approach assigns higher weights to regions with faster data changes and low-value areas, thereby improving the model’s prediction accuracy in these regions. Thus, the flexible weighting mechanism effectively enhances the model’s inference performance in diverse value intervals and avoids the loss of accuracy caused by the uniform weighting method. The relevant formulas are presented in the following equations:

(1) $$\text{Loss}=\frac{1}{N}\overset{N}{\underset{i=1}{\sum }}\left(w_{\mathrm{grad},i}\cdot w_{\min,i}\cdot {\left(\text{pre}{\text{d}}_{i}-\text{targe}{\text{t}}_{i}\right)}^{2}\right),$$

where *N* is the number of samples, *w*_grad,*i*_ is a weight based on the target gradient to emphasize regions with significant gradient variations. *w*_min,*i*_ is a weight based on the target value to emphasize regions with transmission spectral values less than 0.4. The pred_*i*_ is the predicted value of the *i*th sample, and the target_*i*_ is the actual value of the *i*th sample.

The inverse neural network model illustrated in Figure 3(b) aims to infer nine structural parameters inversely from 120 spectral data points. It employs a combined architecture of one-dimensional convolutional layers and fully connected regression modules. The convolutional layer (magenta rectangle in Figure 3), serving as the core feature extraction module, is integrated with batch normalization (yellow rectangle) and the ReLU activation function. This combination enhances the model’s nonlinear representation capabilities through multiple convolutional layers. Subsequently, the MaxPooling layer (dark green rectangle) reduces the spatial dimensions of the extracted features, enabling the network to capture more abstract and high-level representations and accurately predict the target parameters. This convolutional design empowers the model to capture important local features from the spectral data, thus providing stronger feature support for subsequent regression prediction. Following feature extraction, the output is spread and fed into the fully connected layer for regression prediction. Again, this network uses the ReLU activation function and Dropout regularization after each layer, aiming to capture global features of the data further and avoid overfitting phenomena. Meanwhile, the introduction of LayerNorm (gray rectangle in Figure 3) helps to improve the stability of the training process. The detailed input and output dimensions of the inverse prediction model are shown in Table 3.

**Table 3:** Inverse neural network size input and output table.

Block	Size-in	Size-out
Conv block 1	Input size	32 × 120 × 1
PoolDrop block 1	32 × 120 × 1	32 × 60 × 1
Conv block 2	32 × 60 × 1	64 × 60 × 1
PoolDrop block 2	64 × 60 × 1	64 × 30 × 1
Conv block 3	64 × 30 × 1	128 × 30 × 1
MaxPool layer	128 × 30 × 1	128 × 10 × 1
Squeeze layer	128 × 10 × 1	1,280 × 1
FC block 1	1,280 × 1	256 × 1
FC block 2	256 × 1	128 × 1
FC block 3	128 × 1	64 × 1
FC layer	64 × 1	Output size

To ensure that the network is sufficiently trained, we employ AdamW, the optimizer of choice in modern deep learning practice, which achieves a purer regularization effect by decoupling weight decay and adaptive learning rate updating, resulting in better generalization performance than standard Adam in most cases. A customized loss function Total_loss was developed based on a combination of L1_loss and Weighted_MSELoss. The first loss function metric quantifies the absolute error between the predicted value and the target value, allowing the network to focus on regions with minor errors during training; the second metric quantifies the mean squared deviation between each predicted value and the target value, and dynamically adjusts the size of the weights according to the specific importance of each structural parameter. The related formula is shown below:

(2) $$\text{L}1\text{\_}\text{loss}=\frac{1}{N}\overset{N}{\underset{i=1}{\sum }}\left|\text{pre}{\text{d}}_{i}-\text{targe}{\text{t}}_{i}\right|,$$

(3) $$\text{Weighted}\_\text{MSELoss}=\frac{1}{N}\overset{N}{\underset{i=1}{\sum }}{\left(\text{pre}{\text{d}}_{i}-\text{targe}{\text{t}}_{i}\right)}^{2},$$

(4) $$\text{Total}\_\text{loss}=\alpha \cdot \text{L}1_{\text{loss}}+\left(1-\alpha \right)\cdot \text{Weighted}\_\text{MSELoss},$$

where *α* is a balancing parameter that controls the contribution of the L1_loss and the Weighted_MSELoss, which balances the contribution of different errors to the final result.

**Figure 4:**
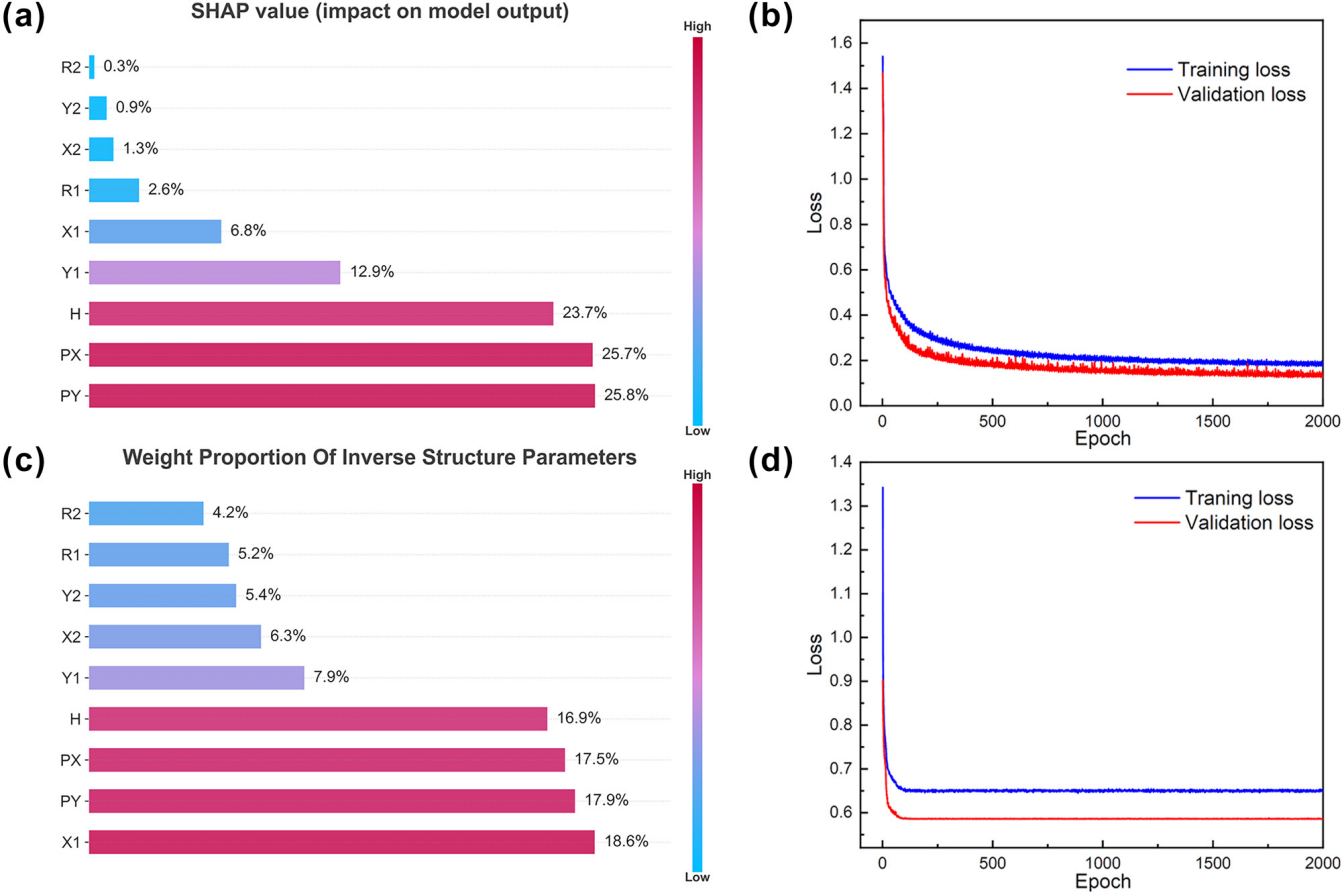
Plot of forward and inverse neural network training results. (a) Weight share of forward neural network structural parameters under the SHAP model. (b) Forward neural network loss function plot. (c) Weight share of the inverse neural network structure parameters in the last output layer. (d) Inverse neural network loss function plot.

To quantify the weight share of each parameter in the two networks with the training of the network, the introduction of the SHAP (Shapley Additive exPlanation) [50] framework can well reflect the influence of the features in each sample and Figure 4(a), the weight share of the structural parameters of the forward neural network shows that the rotational angle has a smaller weight, which indicates that the rotational angle has a weak influence on the final structural output is weakly influenced. Figure 4(c), on the other hand, shows the weight share of the inverse neural network in the last output layer, indicating that the rotation angle contributes less to the spectral data, thus explaining why the rotation angle disappears after optimization. Figure 4(b) shows the loss function plot of the forward neural network, where the loss value gradually decreases with training, indicating that the forward neural network achieves a good fit in mapping spectral data to structural parameters. Figure 4(d) shows the loss function plot of the inverse neural network, and the loss value also gradually decreases with the advancement of the training process, which verifies the accuracy of the inverse neural network in inferring the structural parameters from the spectral data in reverse. The two-loss function plots show that both forward and inverse neural networks exhibit sound fitting effects during the training process, and the continuous reduction of the loss function value indicates that the model is continuously optimized to improve the prediction accuracy.

### 2.3 Structural analysis

**Figure 5:**
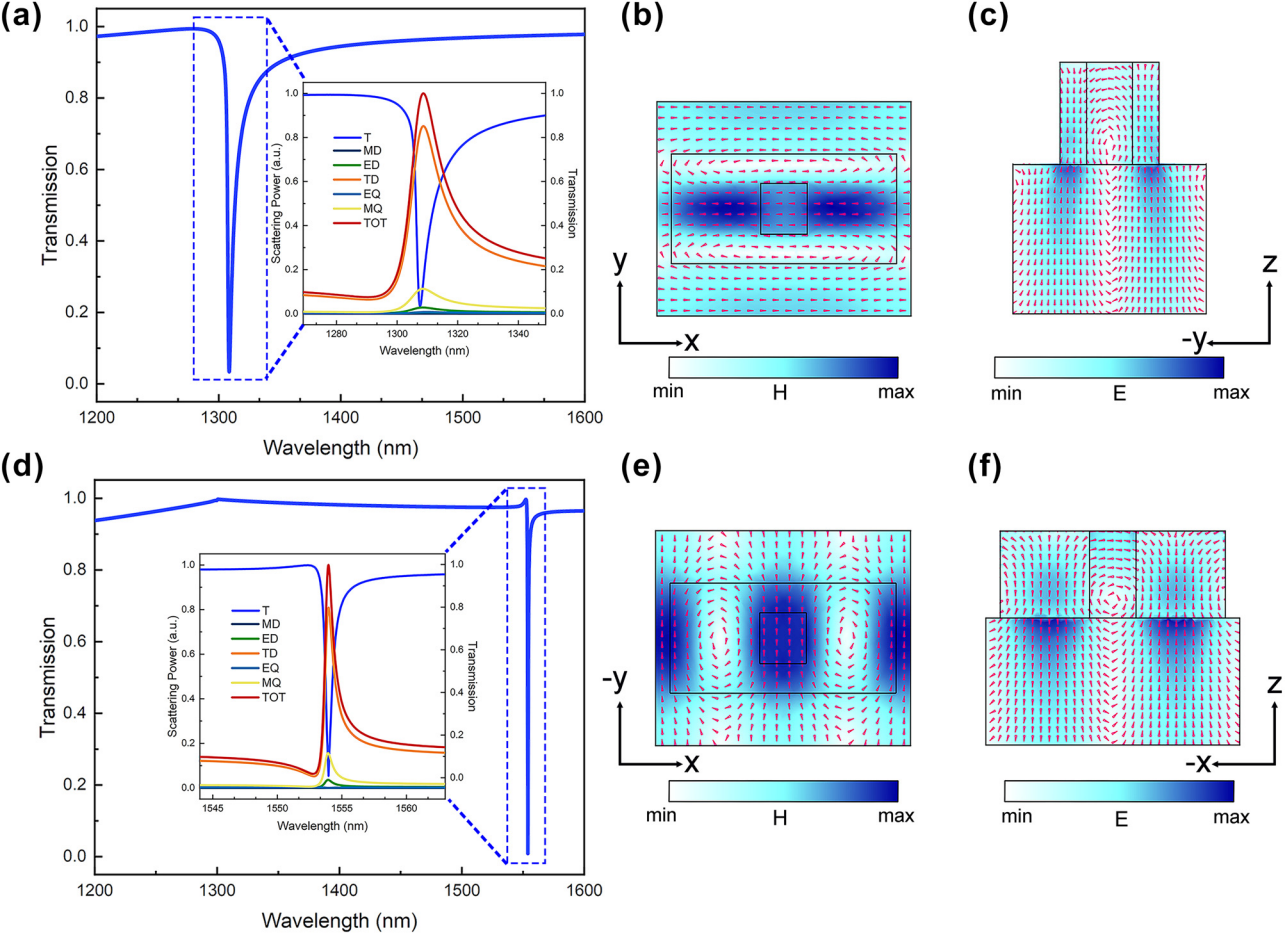
Transmission spectrum multipolar decomposition with electromagnetic field analysis in dual polarization. (a) Transmission spectrum of the 1,200–1,600 nm band with multipolar decomposition at 90° polarization. (b) Magnetic field distribution of the metasurface structure at the transmission peak in the *x*–*y* plane under 90° polarization. (c) Electric field distribution in the *y*–*z* plane of the metasurface structure at the transmission peak under 90° polarization. (d) Transmission spectrum of the 1,200–1,600 nm band with multipolar decomposition at 0° polarization. (e) Magnetic field distribution in the *x*–*y* plane of the metasurface structure at the transmission peak under 0° polarization. (f) Electric field distribution in the *x*–*z* plane of the metasurface structure at the transmission peak at 0° polarization.

With the previous deep learning optimization iterative structure, a rectangular nano-block with a longer length in the *y*-direction and a rectangular vacancy in the middle is finally obtained. As shown in Figure 5(a) and (d), with independent transmission peaks in the O-band and C-band, respectively, under orthogonally polarized incidence, which is perfectly suitable for dual-band switching applications in optical communication. To reveal the resonance mode mechanism involved in the transmission peaks and the trapping ability of this metasurface structure for electric and magnetic fields, the relevant transmission spectra, multipolar decomposition [51], and vector and field strength distributions of the electromagnetic fields are shown in Figure 5. Analysis of the transmission spectra and multipole decomposition for the 1,200–1,600 nm bands under 90° and 0° polarized light (Figure 5(a) and (d)) reveals that the toroidal dipole (TD) response primarily contributes to the observed transmission peaks. Further analysis of the magnetic field vector distributions in Figure 5(b) and (e) show distinct directional dependence on the incident polarization. Further examination of the magnetic field vector distributions in Figure 5(b) and (e) show distinct directional dependence on the incident polarization. The magnetic field vector in Figure 5(b) (90° polarization) is predominantly concentrated along the *x*-direction, while in Figure 5(e) (0° polarization), it is mainly focused along the *y*-direction. This polarization-dependent behavior correlates with the formation of two oppositely rotating toroidal magnetic field distributions in the *x*–*y* plane. This specific magnetic field configuration directly facilitates the formation of aligned head-to-tail electric dipole moments, as visualized in Figure 5(c) and (f), thereby supporting the accuracy of the multipole decomposition analysis.

Under 90° polarized light, the magnetic field distribution is localized in the left and right sides of the nano-blocks in the *x*-direction, as shown in Figure 5(b), and the electric field is localized at the junction of the nano-blocks and the substrate, as shown in Figure 5(c). Under 0° polarized light irradiation, the magnetic field distribution, as shown in Figure 5(e), is localized in the air gap near the air holes and the edge of the metasurface structure. The electric field, as shown in Figure 5(f), is localized in the nano-blocks and the junction of the substrate. Consequently, this analysis of the localized electromagnetic fields effectively explains the enhanced light–matter interactions at the nanoscale within the metasurface structure. The demonstrated capability for polarization-dependent field confinement provides a unique advantage for this structure in optical applications [52], [53], [54], particularly polarization-switching communication bands.

## 3 Results

**Figure 6:**
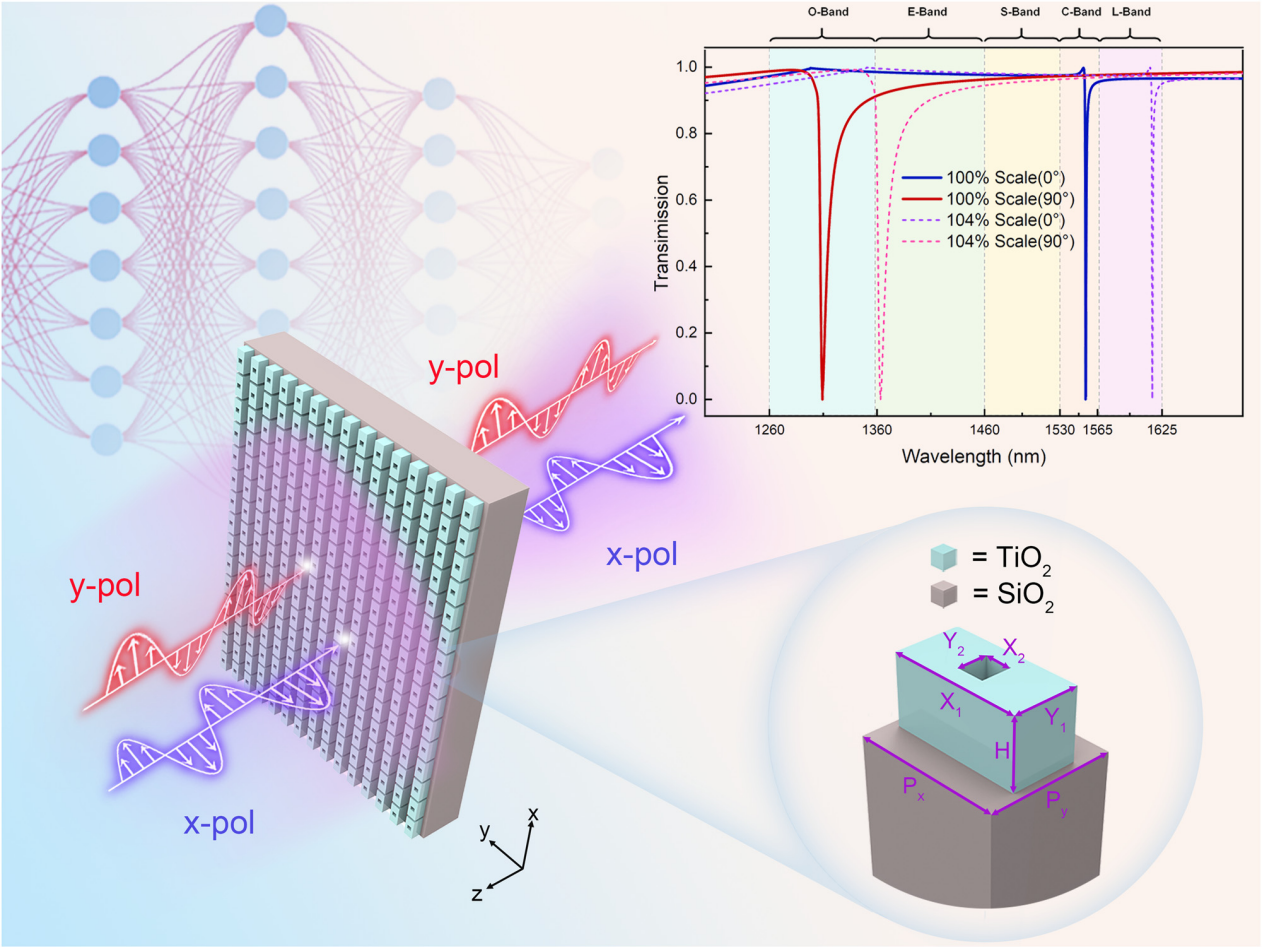
Schematic diagram of the metasurface function for polarization modulation to achieve polarization-dependent switching in dual-band for optical communication. It shows that when subjected to incident light with *x*-direction and *y*-direction polarization, polarized light exhibits distinct transmission peaks in the C-band and O-band. When the metasurface unit cell is scaled proportionally to 104 %, the transmission peaks shift to the E-band and L-band. The parameters of the metasurface unit cell are *P*_*x*_ = 1,076 nm for the *x*-direction, *P*_*y*_ = 906 nm for the *y*-direction, *X*_1_ = 954 nm for the width of the TiO_2_ nano-blocks, *Y*_1_ = 464 nm for the length, *X*_2_ = 197 nm for the width of the square holes, *Y*_2_ = 215 nm for the length, *H* = 479 nm for the height, and the angle of rotation is 0°.

After the iterative process and analysis described above, we finally obtained the metasurface structure as shown in the functional demonstration in Figure 6. This structure relies on modulating linearly polarized light in the *x*–*y* plane to generate transmission peaks at the C-band and O-band. Moreover, upon scaling the metasurface parameters proportionally, the transmission peaks shift proportionally while perfectly preserving their waveform characteristics. When scaled to 104 %, the structure successfully excites independent transmission peaks in both the E-band and L-band, signifying that its switching function has shifted from the original O/C-band to the E/L-band. The function of this translational transmission peak demonstrates that our proposed research approach – “identifying the initial structure in the target band followed by deep learning optimization to achieve band switching” – possesses strong universal applicability.

We have significantly improved the efficiency of structural design by combining the idea of nano-simulation design with deep learning methods, which effectively improves the utilization efficiency of the corresponding spectral bands in the communication system, and nicely enhances the switching function of this metasurface. Through training on numerous data samples, deep learning employs inverse design to automatically identify the most suitable design parameters (e.g., period, meta-atom height, and size) to ensure optimal optical transmission characteristics within a target wavelength range. This process significantly shortens the design cycle while improving the tuning accuracy of the transmission peaks. We have selected titanium dioxide as the primary material for metasurface meta-atomic structures since the relatively high refractive index and low optical loss make it suitable for enhanced optical modulation. Meanwhile, the SiO_2_ substrate provides stabilizing support to ensure the mechanical stability of the structure. Importantly, this structure offers excellent theoretical performance while maintaining practical fabrication feasibility. The simple structure (consisting of air and TiO_2_ rectangular blocks) obtained through deep learning optimization can effectively adapt to existing micro/nanofabrication techniques, avoiding the production challenges associated with complex structures. The anisotropic nanostructure is used to support different polarizations of the incident light source to produce different responses; the hole dug in the middle of the structure is used to shrink the FWHM of the transmission peaks to increase the *Q*-value, achieving better independent switching between the two communication bands throughout the system. Although this metasurface has achieved communication band switching to a certain extent, the transmission peaks in the spectral image have not yet reached their ideal state FWHM levels. Moreover, this work has conducted fundamental research on the realization of future metasurfaces, enabling the switching of arbitrary communication bands, and has contributed to pioneering work in the unexplored small field.

## 4 Discussion

This work proposes a polarization regulation method of metasurface based on deep learning optimization design, which successfully realizes the polarization-dependent switching of dual-band for communication. Compared to traditional approaches, the deep learning-optimized metasurface proposed in this study achieves O/C band selection through simple polarization switching. This fundamentally resolves the inherent bottlenecks of conventional technologies in processing speed, integration density, and functional flexibility. It transforms the band-switching mechanism from slow mechanical motion to polarization modulation with light-speed response, achieving a significant leap in speed. The core design innovation lies in the inverse design framework: after initial structural screening, a deep learning model precisely optimizes the metasurface geometry to achieve polarization-dependent band switching. This approach not only enhances design efficiency by orders of magnitude but also significantly improves system performance metrics. Material selection of TiO_2_ nanostructures on a SiO_2_ substrate ensures both high-efficiency optical modulation and mechanical robustness. The fabrication feasibility is further guaranteed by the materials’ compatibility with established industrial semiconductor processes. Multipolar decomposition analysis and electromagnetic field simulations elucidate the underlying resonance mechanisms under different polarizations, confirming the capability for dynamic band switching. This optical metasurface, capable of changing communication bands by polarized light, can increase bandwidth and data transmission rate, especially in spectrum multiplexing systems with promising applications. Furthermore, with its flexibility in polarization state tuning, the technology can also be widely used in smart optical devices, such as polarization-dependent optical filters, optical switches, and tunable optical sensors in the future. More importantly, this deep learning-driven inverse design paradigm demonstrates how to create multifunctionality for a single metasurface device, laying a solid foundation for developing multifunctional, ultra-compact, and intelligent next-generation photonic systems.
